# Primary Skull Base Chondrosarcomas: A Systematic Review

**DOI:** 10.3390/cancers13235960

**Published:** 2021-11-26

**Authors:** Paolo Palmisciano, Ali S. Haider, Mohammadmahdi Sabahi, Chibueze D. Nwagwu, Othman Bin Alamer, Gianluca Scalia, Giuseppe E. Umana, Aaron A. Cohen-Gadol, Tarek Y. El Ahmadieh, Kenny Yu, Omar N. Pathmanaban

**Affiliations:** 1 Department of Neurosurgery, Trauma Center, Gamma Knife Center, Cannizzaro Hospital, 95126 Catania, Italy; umana.nch@gmail.com; 2 Texas A&M University College of Medicine, Houston, TX 77030, USA; aralam09@gmail.com; 3 Neurosurgery Research Group (NRG), Student Research Committee, Hamadan University of Medical Sciences, Hamadan 65141, Iran; mmsabahi1996@gmail.com; 4 Emory University School of Medicine, Atlanta, GA 30322, USA; dominicnwagwu@gmail.com; 5 Department of Neurosurgery, King Abdullah International Medical Research Center, Riyadh 11451, Saudi Arabia; oabinalamer@gmail.com; 6 Department of Neurosurgery, Highly Specialized Hospital of National Importance “Garibaldi”, 95126 Catania, Italy; gianluca.scalia@outlook.it; 7 Department of Neurological Surgery, Indiana University School of Medicine, Indianapolis, IN 46077, USA; cohen@nsatlas.com; 8 Department of Neurosurgery and Brain Metastasis Center, Memorial Sloan Kettering Cancer Center, New York, NY 10065, USA; telahmadieh@gmail.com (T.Y.E.A.); yuk2@mskcc.org (K.Y.); 9 Manchester Skull Base Unit, Department of Neurosurgery, Manchester Centre for Clinical Neurosciences, Geoffrey Jefferson Brain Research Centre, Salford Royal Hospital, Manchester Academic Health Science Centre, University of Manchester, Manchester M13 9WL, UK; omar.pathmanaban@manchester.ac.uk

**Keywords:** chondrosarcoma, endoscopy, radiation oncology, skull base oncology, systematic review

## Abstract

**Simple Summary:**

Primary skull base chondrosarcomas (SBCs) may carry significant tumor-burden by causing severe cranial nerve neuropathies. Current treatment strategies mainly focus on surgical resection and radiotherapy protocols, with a wide range of findings in terms of efficacy and safety. The aim of our systematic review was to comprehensively analyze the current literature on primary SBCs, describing clinical and radiological characteristics, available management strategies, treatment outcomes, and prognoses. We found that most primary SBCs show benign slow-growing patterns but may cause neurological deficits by compressing critical neurovascular structures. Open surgical approaches favor maximal resection with acceptable complication rates, but only a few studies reported the use of newer endoscopic approaches. Proton-based, photon-based, and carbon-based radiotherapy protocols may also allow safe and effective local tumor control as adjuvant treatments or stand-alone strategies in patients not eligible to undergo surgery. Overall, primary SBCs’ prognoses proved to be favorable and comparable to benign skull base neoplasms.

**Abstract:**

Background: Primary skull base chondrosarcomas (SBCs) can severely affect patients’ quality of life. Surgical-resection and radiotherapy are feasible but may cause debilitating complications. We systematically reviewed the literature on primary SBCs. Methods: PubMed, EMBASE, Scopus, Web-of-Science, and Cochrane were searched following the PRISMA guidelines to include studies of patients with primary SBCs. Clinical characteristics, management strategies, and treatment outcomes were analyzed. Results: We included 33 studies comprising 1307 patients. Primary SBCs mostly involved the middle-fossa (72.7%), infiltrating the cavernous-sinus in 42.4% of patients. Cranial-neuropathies were reported in 810 patients (62%). Surgical-resection (93.3%) was preferred over biopsy (6.6%). The most frequent open surgical approaches were frontotemporal-orbitozygomatic (17.6%) and pterional (11.9%), and 111 patients (21.3%) underwent endoscopic-endonasal resection. Post-surgical cerebrospinal-fluid leaks occurred in 36 patients (6.5%). Radiotherapy was delivered in 1018 patients (77.9%): photon-based (41.4%), proton-based (64.2%), and carbon-based (13.1%). Severe post-radiotherapy complications, mostly hypopituitarism (15.4%) and hearing loss (7.1%) were experienced by 251 patients (30.7%). Post-treatment symptom-improvement (46.7%) and reduced/stable tumor volumes (85.4%) showed no differences based on radiotherapy-protocols (*p* = 0.165; *p* = 0.062). Median follow-up was 67-months (range, 0.1–376). SBCs recurrences were reported in 211 cases (16.1%). The 5-year and 10-year progression-free survival rates were 84.3% and 67.4%, and overall survival rates were 94% and 84%. Conclusion: Surgical-resection and radiotherapy are effective treatments in primary SBCs, with acceptable complication rates and favorable local tumor control.

## 1. Introduction

Chondrosarcomas comprise a heterogeneous group of malignant tumors originating from chondroid cells throughout the appendicular and axial skeleton. The skull base is involved in approximately 1% of cases, with skull base chondrosarcomas (SBCs) accounting for 0.15% of all intracranial neoplasms [1,2]. Despite the proven association with Ollier’s and Maffucci’s diseases, SBCs mostly arise de novo [3]. Their pathogenesis appears to be linked to the endochondral ossification of the skull base synchondroses, as most tumors involve the clivus and the temporo-occipital junction [4,5]. Amongst the histopathological subtypes, conventional chondrosarcomas represent the vast majority and are classified into WHO grades I (well-differentiated), II (intermediate), and III (poorly differentiated) [6,7].

While low-grade SBCs may be indolent and slow-growing, poorly differentiated neoplasms are highly invasive, destructive, and may metastasize. Symptoms derive from the direct compression of cranial nerves and neurovascular structures at the base of the skull, with headache and diplopia being the most frequent [5,8]. Computer tomography (CT) scans can evaluate bone invasion and destruction, but magnetic resonance imaging (MRI) provides better delineation of soft tissue involvement in skull base chondromas [8,9,10]. The main goals of surgery are tissue diagnosis and maximal safe resection, in order to prevent neurological deterioration, optimize conditions for dose-escalated adjuvant radiotherapy, and improve survival. Gross total resection is safely feasible in some cases, but has higher risks of postoperative neuropathies and vascular injuries [11,12]. Thus, adjuvant radiotherapy is often administered to treat any residual disease following safe maximal cytoreduction, further enhancing local tumor control and survival [11,13]. Novel proton-based and carbon ion-based radiotherapy modalities have also been studied to focus high dose radiation beams to selected tumor targets, reducing radiation-induced toxicities to the normal brain tissue [14,15].

Due to the rare incidence of SBCs, our understanding of the natural history and management of this disease is mostly derived from a relatively small number of studies with heterogeneous clinical characteristics and treatments [16,17]. The aim of this systematic review, therefore, is to comprehensively summarize the demographics, clinical features, and management strategies in patients with primary SBCs.

## 2. Materials and Methods

### 2.1. Literature Search

A systematic review was performed upon the Preferred Reporting Items for Systematic Reviews and Meta-Analyses (PRISMA) statement [18] and registered to PROSPERO (ID: 287304). PubMed, EMBASE, Scopus, Web of Science, and Cochrane were searched from inception to 6 May 2021 using the combination of the Boolean operators “OR” and “AND” and the search terms: “skull”, “base”, and “chondrosarcoma”. Studies were exported to Mendeley, and duplicates were removed.

### 2.2. Study Selection

A priori inclusion and exclusion criteria were defined. Studies were included if they were: (1) studies including 5 or more patients aged 18 years or older with histologically confirmed primary chondrosarcomas involving the base of the skull; (2) studies reporting data on clinical features, treatment strategies, post-treatment outcomes; (3) written in English. Studies were excluded if they were: (1) meta-analyses, literature reviews, technical notes, editorials, or books; (2) studies involving patients with secondary metastatic chondrosarcomas affecting the skull base; (3) studies with an unclear distinction between patients with skull base chondrosarcomas and patients with skull base chordomas; (4) studies with insufficient clinical data, lacking 2 or more of: patients’ demographics, clinical characteristics, management strategies, and post-treatment outcomes.

Two authors (M.S. and P.P.) independently screened titles and abstracts of all collected studies, and then assessed full texts of articles that met inclusion criteria. A third author (A.S.H.) settled any disagreements. Eligible studies were included based on the predefined criteria and references were screened to retrieve additional relevant articles.

### 2.3. Data Extraction

Data were extracted by one author (C.D.N.) and then confirmed independently by two additional authors (P.P. and O.B.A.). Missing data were either not reported or not differentiable from other non-relevant data. Extracted data included: authors, year, sample size, age, gender, syndromes, tumor laterality and location, extra/intra-axial structures involved, symptoms, cranial nerve neuropathies, tumor size, histopathological WHO grade and type valid at the time of tumor diagnosis/treatment or study publication, biopsy or surgical resection, extent-of-surgery, surgical approach, post-surgical complications, radiotherapy protocols, severe post-radiotherapy complications, clinical and radiological outcomes, recurrence, progression-free survival (PFS), overall survival (OS), and survival status. The extent-of-surgical resection was defined as “gross total resection” for 100% tumor resection, “subtotal resection” for 80–99% resection, and “partial resection” for <80% resection. Post-surgical complications were divided into “transient”, if self-resolving or treated with only medical therapy, and “persistent”, if untreatable. Clinical outcomes and radiological responses were assessed at 6-months post-treatment or at the last available follow-up.

### 2.4. Data Synthesis and Quality Assessment

Primary outcomes of interest were the clinical characteristics, management strategies, and post-treatment outcomes of patients with primary SBCs. The level of evidence of each article was evaluated upon the 2011 Oxford Centre For Evidence-Based Medicine guidelines [19]. Meta-analysis was precluded because all included studies had level IV of evidence and hazard ratios could not be deducted. The risk of bias of each article was independently assessed by two authors (P.P. and O.B.A.) using the Joanna Briggs Institute checklists for case series [20].

### 2.5. Statistical Analysis

The software SPSS V.25 (IBM Corp, Armonk, New York, NY, USA) was used for all statistical analyses. A two-tailed *p*-value < 0.05 was considered significant for all tests. Continuous variables are summarized as medians or means and ranges, while categorical variables are reported as frequencies and percentages. Rates of post-treatment outcomes and complications were compared using χ2 and Fisher exact tests. The time intervals between surgery and SBCs recurrence (PFS curve) or death (OS curve) were estimated using the Kaplan-Meier method, and the survival analyses were conducted using the log-rank test.

## 3. Results

### 3.1. Study Selection

**Figure 1:**
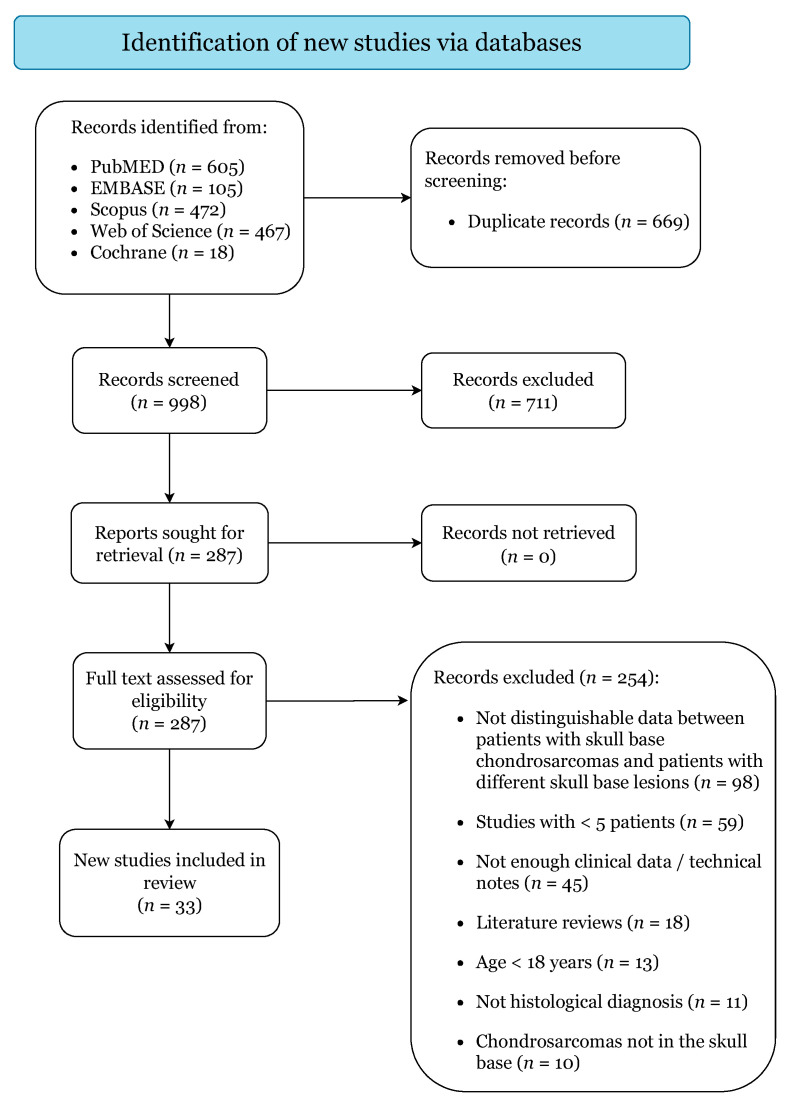
PRISMA 2020 Flow-Diagram.

Figure 1 illustrates the study selection process. The initial search yielded 1667 citations (PubMed: 605; EMBASE: 105; Scopus: 472; Web of Science: 467; Cochrane: 18). A total of 33 case series were finally included upon the pre-specified criteria, categorized as level IV of evidence (Table S1) [4,13,15,16,17,21,22,23,24,25,26,27,28,29,30,31,32,33,34,35,36,37,38,39,40,41,42,43,44,45,46,47,48]. Quality assessment returned a low risk of bias for all included studies (Table S2).

### 3.2. Demographics and Clinical Characteristics

Table 1 shows the demographics and anatomical features of all 1307 included patients. Patients were mostly female (53%) with a median age of 42.5 years (range, 18–85). Ollier’s disease was reported in twelve patients (0.9%) and Maffucci’s disease in three (0.2%). Most SBCs occurred in the middle fossa (72.7%) involving the petrous bone (37.8%), the clivus (23.5%), and the petroclival synchondrosis (20.2%). Less frequently, tumors infiltrated the anterior fossa (13.1%), involving the supra/parasellar region (7.4%) and/or the orbit (2.3%), or the posterior fossa (20.9%), extending to the jugular foramen (4.4%) and/or to the foramen magnum/occipital bone (1.7%). SBCs invaded the cavernous sinus in 42.4% of patients and caused extra-axial compression of the brainstem and the optic apparatus in 49.7% and 38.4% of cases, respectively. Intra-axial invasion of the temporal lobe was reported in three patients with recurrent SBCs (0.2%). The median tumor volume was 24.3 cm^3^ (range, 0.9–88.4).

Most patients experienced various degrees of debilitating symptoms, especially diplopia (29.2%) and headache (21.8%), for a median duration of 16 months (range, 0.1–312) (Table 2). Of note, only 51 patients (6.3%) were asymptomatic, with SBCs detected at incidental radiological exams. Cranial nerve neuropathies were reported in 810 patients (62%), most commonly involving the fifth (19.4%) and the sixth (31.6%) cranial nerves, and multiple cranial nerves in 161 cases (20.2%). Hypopituitarism was also recorded in 29 patients (3.6%). At histopathology, conventional SBCs were the most common (84.5%), followed by myxoid (7.6%), mesenchymal (5.5%), and undifferentiated (0.3%) subtypes. WHO grades were reported in 909 patients with conventional SBCs: tumors with low/I grade were the most common (59.9%), followed by tumors with II/intermediate grade (37.6%) and III/high grade (2.5%).

### 3.3. Management Strategies

Management strategies are reported in Table 3. Only 87 patients (6.6%) received biopsy, while 1220 patients (93.3%) underwent surgical resection: open in 1092 (89.5%), endoscopic in 111 (9.1%), and combined in 17 (1.4%). Gross total, subtotal, and partial tumor resection were obtained in 37.8%, 45.7%, and 16.5% of patients, respectively. The most frequent open surgical approaches were the frontotemporal-orbitozygomatic (17.6%) and the pterional (11.9%), while the endonasal transsphenoidal route was pursued in all patients undergoing endoscopic resection.

A total of 1018 patients (77.9%) received radiotherapy after histological confirmation of SBCs. Conventional photon-based radiotherapy was delivered in 421 patients (32.2%) with a median dose of 55 Gy (range, 6.5–70): external beam radiotherapy in 249 (59.1%), Gamma Knife in 82 (19.5%), intensity-modulated radiotherapy in 40 (9.5%), linear accelerator (LINAC) in 34 (8.1%) and Cyber Knife in 16 (3.8%). Proton-based radiotherapy was delivered in 654 patients (50%) with a median dose of 70 GyE (range, 12–76), and carbon ion-based radiotherapy in 133 patients (10.2%) with a median dose of 60 GyE (range, 57–69).

### 3.4. Treatment Outcomes, Complications, and Survival

Table 4 shows post-treatment outcomes and complications. A total of 36 patients (6.5%) experienced post-surgical cerebrospinal fluid (CSF) leaks, which required a second operation for repairing the dural defect with autologous fat and muscle grafts. Transient post-surgical complications were described in 88 patients (15.8%), mostly new self-resolving cranial nerve neuropathies (10.5%) and meningitis (2.9%). Persistent post-surgical complications were reported in 59 patients (10.6%), mostly new untreatable cranial nerve neuropathies (6.7%) and intracerebral hemorrhages (1.4%). A total of 251 patients (30.7%) experienced severe debilitating post-radiotherapy complications, mostly hypopituitarism (15.4%), hearing loss (7.1%), and brain/spinal cord radiation necrosis (3.7%). With regards to the radiation-induced onset of new locoregional neoplasms, we found two brainstem gliomas (0.2%) [36,41], one supratentorial glioblastoma (0.1%) [41], and one convexity meningioma (0.1%) [17], with no cases of new post-radiation sarcomas nor dedifferentiation of previous chondrosarcomas reported across our included studies. Rates of severe complications were statistically higher in patients receiving radiotherapy compared to patients undergoing tumor resection (including CSF leaks and persistent complications) (*p* < 0.001).

At post-treatment follow-up, symptomatic improvement was described in 46.7% of patients, with no significant differences based on the type of radiotherapy protocol (*p* = 0.165). At post-radiotherapy follow-up, most lesions showed radiological stable volumes (58.3%) for tumor shrinkage (27.1%), with few cases of increased tumor volumes (14.6%). No significant differences in radiological responses were found based on the type of radiotherapy protocol (*p* = 0.062).

**Figure 2:**
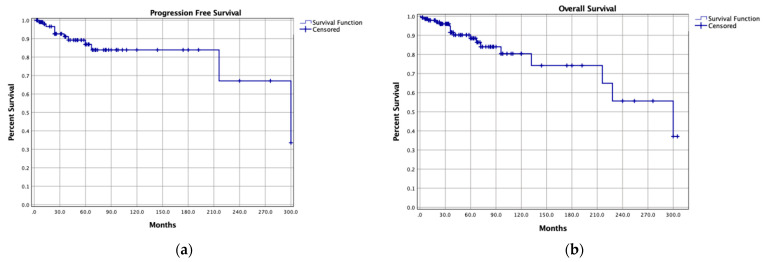
Kaplan-Meier curves of (**a**) progression-free survival and (**b**) overall survival of the pooled cohort.

The median follow-up time was 67 months (range, 0.1–376). Local SBCs recurrences were reported in 211 patients (16.1%) and distant metastases in seven (0.5%), with 5-year and 10-year PFS rates of 84.3% and 67.4%, respectively. In most cases, treatment strategies of local SBCs recurrences were not described (41.2%), but, among described cases, most local recurrences were treated with surgical resection alone (15.6%) or with adjuvant radiotherapy (8.1%), or with radiotherapy alone (13.7%). Most patients were alive at last follow-up (88.8%), with 5-year and 10-year OS rates of 94% and 84%, respectively (Figure 2).

## 4. Discussion

Primary SBCs are uncommon but challenging entities with a major impact on patients’ quality of life. Current management strategies are aimed at relieving symptoms, tumor control, and protecting functional status, but related complications need to be considered. In this review, we provide a comprehensive summary of the current literature regarding primary SBCs, analyzed within the context of other bone chondrosarcomas and skull base neoplasms.

In our cohort, primary SBCs’ incidence peaked between the fourth and sixth decades of life, similarly to previous reports on spinal and laryngeal chondrosarcomas from national cancer databases and systematic reviews [49,50,51]. However, while spinal chondrosarcomas mostly affect men, primary SBCs showed no gender predilection, probably suggesting underlying molecular differences that deserve further evaluation [50,51]. Chondrosarcomas are deemed to originate from chondroid cells and are grouped in different histological subtypes based on cellular appearance [7]. Regardless of the anatomical location, conventional chondrosarcomas represent the vast majority, mostly of low/intermediate grade with slow growth patterns; the other less common subtypes, such as mesenchymal, identify high-grade aggressive variants [49,51,52,53]. Similarly, most tumors in our review were conventional SBCs (84.5%) of low (59.9%) or intermediate grade (37.6%), while mesenchymal SBCs showed the worst prognoses [38,39]. Chondrosarcomas may also derive from the de-differentiation of primary enchondromas, which may occur more frequently in patients with congenital systemic enchondromatosis [54,55]. Indeed, we found that some patients with primary SBCs had underlying Ollier’s (0.9%) and Maffucci’s (0.2%) syndromes, in line with the current literature on axial chondrosarcomas [51,52]. In these cases, management strategies are complex and multi-disciplinary treatment approaches are recommended [55].

Most patients with primary SBCs experienced mild and slowly progressing symptoms for a median duration of 16 months before requesting medical assistance. Similar findings have been reported in patients with spinal and laryngeal chondrosarcomas, likely suggesting that these entities mostly carry indolent clinical courses following their slow growth patterns [49,50]. Still, the acute onset of symptoms in some patients may suggest that some primary SBCs may remain asymptomatic until the occurrence of severe neurologic compromise, while other SBCs, especially high grade or non-conventional, may show rapid tumor progression and neurological impairments [23,39,48]. Symptoms of primary SBCs reflect their location within the skull base and their proximity to critical neurovascular structures [17,36]. We found that most primary SBCs occurred in the middle fossa, mainly involving the petrous bone (37.8%), the clivus (23.5%), and/or the petroclival synchondrosis (20.2%), and also compressing the brainstem (49.7%) and/or invading the cavernous sinus (42.4%). Hence, tumors primarily caused direct compression and injury of the sixth cranial nerve (31.6%), leading to diplopia (29.2%) in most patients. As described by Feuvret et al. [36], primary SBCs may also invade the anterior fossa, mostly causing vision impairment and hypopituitarism by directly compressing the optic apparatus or the pituitary stalk. However, such symptoms are non-specific and may also occur in patients with skull base chordomas and other sarcomas [8,56,57]. Likewise, radiological features of chordomas and SBCs may overlap, with similar localization and bone destructive patterns [58]. Histological confirmation is thus needed for accurate diagnosis and treatment planning.

In view of the complex anatomy of primary SBCs, management strategies require multidisciplinary approaches encompassing neurosurgery, otolaryngology, maxillofacial surgery, ophthalmology, and radiation oncology [5,56,59]. A stand-alone biopsy is rarely pursued, carrying surgical risks comparable to resection whilst providing limited clinical benefit [23,24,36,39]. Tumor resection is preferred, having both a diagnostic and therapeutic role. As described for skull base chordomas and osteosarcomas, the selection of the best surgical approach depends on the tumor’s bony epicenter and extension, aimed at safely exposing the lesion and the involved cranial nerves [8,56,57,60,61]. In our cohort, the endoscopic transnasal and the open frontotemporal-orbitozygomatic approaches were the most common, better addressing tumors involving the petrous apex and upper clivus and anteriorly invading the cavernous sinuses [35,39,40,43]. By contrast, retro-sigmoid and trans-petrosal approaches were performed to manage less frequent posterior fossa primary SBCs extending to the internal acoustic canal [29,31]. Of note, staged procedures were pursued in patients with large tumors not amenable to a single surgical resection [4,13,36]. With regards to the extent-of-surgery, different authors argued for aggressive gross total resection or safe cytoreduction with adjuvant radiotherapy, weighting surgical risks against benefits in terms of local tumor control and survival [15,25,27]. More recently, Patel et al. [11] analyzed the United States national cancer database and found no significant differences between partial and radical SBCs resection, but advocated functional-sparing subtotal resection followed by adjuvant radiotherapy, achieving satisfactory outcomes without sacrificing patients’ functional status. When subtotal resection is planned however, it must be done with the optimal parameters for radiotherapy in mind, to ensure the minimal number of surgeries possible without compromising optimal radiation dosing or increasing radiation complications. A minimum of 3 mm clearance from brainstem and 5 mm from optic apparatus are important guiding principles.

Adjuvant radiotherapy has emerged as a cornerstone treatment in the management of skull base chordomas and sarcomas, mainly directed against unresectable tumor portions adherent to critical neurovascular structures [8,56,57]. Although bone chondrosarcomas and SBCs are generally considered to be radioresistant due to their slow growth patterns, post-resection radiotherapy effectively improves local tumor control, especially in high-grade conventional subtypes or non-conventional subtypes [56,62]. In our cohort, adjuvant radiotherapy was delivered with high total doses due to SBCs’ poor radiosensitivity, similarly to radiotherapy protocols for other skull base sarcomas [12,56]. In patients receiving adjuvant radiation, proton-based particle therapy, alone or combined with photon-based protocols, was preferred mostly because of the favorable dosimetric profile (the Bragg peak effect), which allows the delivery of high doses to precise tumor targets with rapid distal fall-off, likely limiting radiation injuries to critical adjacent brain regions [63,64]. The use of carbon-ion-based radiotherapy has also been described for treating skull base chordomas and chondrosarcomas, but its limited availability in a few select centers precludes a comprehensive understanding [14,27,33].

In patients with primary SBCs, the therapeutic goals focus on clinical improvement and local tumor control maintaining function and quality of life and minimizing treatment-related complications. We report favorable rates of symptomatic improvement and radiological tumor volume shrinking in patients receiving surgery plus adjuvant radiotherapy, with no statistical differences based on types of radiotherapy protocol. Although both treatment strategies carry intrinsic risks of severe adverse events, we found significantly higher rates of radiotherapy-related complications as compared to surgical-related complications [17,36,41]. Our findings likely stem from the fact that adjuvant radiotherapy was mostly delivered to remnant tumors deemed unamenable to surgical resection and adjacent to critical neurovascular structures, such as the brainstem, optic apparatus, and the pituitary gland. In addition, our cases of brain/spinal cord radiation necrosis and radiation-induced neuropathies were comparable with those reported for spinal, sinonasal, and laryngeal chondrosarcomas, presumably due to the high radiation doses required to treat these radioresistant lesions [49,51,52,65]. This dose however underscore the importance of attempting to optimize parameters of resection with adjuvant radiation in mind when subtotal removal is undertaken because disease residual close to brainstem and optic apparatus will alter the efficacy and safety of dose-escalated radiation. Finally, we found that the prognosis of SBCs is favorable, with good 5-year and 10-year rates of local tumor control and survival. Several studies also confirmed that the combination of photon/particle-based radiotherapy to surgical resection significantly improves outcomes of SBCs and other bone chondrosarcomas, achieving 5-year PFS and OS rates of 85–95% [13,17,34,36,49,51]. Hence, maximal safe resection coupled with post-surgery radiotherapy should be recommended in eligible patients, especially with large and complex tumors. Yet, the high morbidity and the declining long-term efficacy of surgery and radiation should be taken into account, encouraging the investigation of potential SBCs biomarkers targeted by genetic and immunotherapy approaches [66,67].

### Limitations

Our review has some limitations. All included studies were retrospective case series exposed to selection bias and published within a 32-year time-period characterized by major updates in the WHO classification of Soft Tissue and Bone Tumors and important advances in surgical and radiotherapy protocols, which may have introduced some confounding variables into our analysis. The assessment of post-treatment clinical improvement and the radiological response was subjective in most studies. Due to the lack of granular data found in the literature, we could not comprehensively assess differences in rates of local tumor control, functional status, and survival between patients receiving adjuvant radiotherapy and patients receiving stand-alone surgery. We also could not compare endoscopic versus open techniques, nor analyze the impact of tumor size, histological grade, and subtypes on patients’ functional and survival outcomes. Finally, as we included only patients with a histologically confirmed diagnosis of primary SBCs.

## 5. Conclusions

Primary SBCs are rare and debilitating neoplasms that often require complex and multidisciplinary treatment planning. Surgical debulking and adjuvant radiotherapy protocols show favorable rates of symptomatic improvement and local tumor control, especially in patients with large tumors not eligible for gross total resection. However, treatment-related adverse events are common, and may severely impact patients’ functional status. Novel patient-tailored systemic therapeutic options deserve further evaluation.

## Figures and Tables

**Table 1:** Summary of demographics and anatomical features of all pooled patients.

Characteristics	Value
Cohort size (no.)	1307
Demographics	
Median age, range (years)	42.5, 18–85
Gender (female)	603 (53%)
Syndromes	No. (%)
Ollier’s	12 (0.9%)
Maffucci’s disease	3 (0.2%)
Laterality (*n* = 55)	No. (%)
Right	29 (52.7%)
Left	10 (18.2%)
Midline	4 (7.3%)
Bilateral	12 (21.8%)
Locations (*n* = 859)	No. (%)
Petrous Bone	325 (37.8%)
Clivus	202 (23.5%)
Petroclival synchondrosis	174 (20.2%)
Sphenoid Bone	103 (12%)
Supra/Parasellar region	64 (7.4%)
Others	188 (21.9%)
Extra-Axial Compression/Invasion (*n* = 630)	No. (%)
Brainstem	313 (49.7%)
Cavernous Sinus	267 (42.4%)
Optic Apparatus	242 (38.4%)
Temporal Lobe Compression	139 (22.1%)
Sphenoid Sinus	51 (8.1%)
Internal Carotid Artery	49 (7.7%)
Others	68(10.8%)
Tumor Size (*n* = 681)	
Median, range (cm^3^)	24.5, 0.9–88.4

**Table 2:** Summary of clinical characteristics of all pooled patients.

Characteristics	Value
Presenting Symptoms (*n* = 811)	No. (%)
Median duration, range (months)	16, 0.1–312
Diplopia	237 (29.2%)
Headache	177 (21.8%)
Vision Impairment	64 (7.9%)
Hearing Loss	41 (5.1%)
Hypopituitarism	29 (3.6%)
Motor Deficits	29 (3.6%)
Vertigo	29 (3.6%)
Others	69 (8.5%)
No Symptoms	51 (6.3%)
Cranial Nerve Neuropathies (*n* = 810)	No. (%)
I	3 (0.4%)
II	28 (3.5%)
III	101 (12.5%)
IV	31 (3.8%)
V	157 (19.4%)
VI	256 (31.6%)
VII	77 (9.5%)
VIII	75 (9.3%)
IX	47 (5.8%)
X	60 (7.4%)
XI	29 (3.6%)
XII	59 (7.3%)
Multiple	164 (20.2%)
Histopathological Types (*n* = 579)	No. (%)
Conventional	501 (86.5%)
Myxoid	44 (7.6%)
Mesenchymal	32 (5.5%)
Undifferentiated	2 (0.3%)
WHO Grade (*n* = 928)	No. (%)
I/Low	556 (59.9%)
II/Intermediate	349 (37.6%)
III/High	23 (2.5%)

**Table 3:** Summary of management strategies of all pooled patients.

Characteristics	Value
Surgery (*n* = 1307)	No. (%)
Biopsy	87 (6.6%)
Surgical Resection	1220 (93.3%)
Open	1092 (89.5%)
Endoscopic	111 (9.1%)
Combined (Open + Endo)	17 (1.4%)
Extent of Surgical Resection (*n* = 776)	No. (%)
Gross Total Resection (100%)	293 (37.8%)
Subtotal Resection (80–99%)	355 (45.7%)
Partial Resection (<80%)	128 (16.5%)
Surgical Approach (*n* = 521)	No. (%)
Endonasal Transsphenoidal	111 (21.3%)
Frontotemporal Orbitozygomatic	92 (17.6%)
Pterional	62 (11.9%)
Infra-Temporal	35 (6.7%)
Sub-Temporal	32 (6.1%)
Trans-Petrosal	32 (6.1%)
Retro-Sigmoid	30 (5.8%)
Sub-Temporal + Infra-Temporal	28 (5.4%)
Sub-Frontal/Bi-Frontal	19 (3.6%)
Fronto-Temporal	14 (2.7%)
Others	61 (11.7%)
Conventional Photon-based Radiotherapy (*n* = 1307)	421 (32.2%)
Median dose (Gy), range	55, 6.5–70
External Beam Radiation Therapy	249 (59.1%)
Gamma Knife	82 (19.5%)
Intensity Modulated Radiation Therapy	40 (9.5%)
Linear Accelerator (LINAC)	34 (8.1%)
Cyber Knife	16 (3.8%)
Proton-based Radiotherapy (*n* = 1307)	654 (50%)
Median dose (GyE), range	70, 12–76
Carbon-based Radiotherapy (*n* = 1307)	133 (10.2%)
Median dose (GyE), range	60, 57–69

**Table 4:** Summary of treatment outcomes of all pooled patients.

Characteristics	Value
Post-Surgical Complications (*n* = 555)	No. (%)
Cerebrospinal Fluid Leak	36 (6.5%)
Transient	88 (15.9%)
Cranial Nerve Neuropathies	58 (10.5%)
Meningitis/Brain Abscess	16 (2.9%)
Aphasia	4 (0.7%)
Hearing Impairment	4 (0.7%)
Others	6 (1.1%)
Persistent	59 (10.6%)
Cranial Nerve Neuropathies	37 (6.7%)
Intracerebral Hemorrhage	8 (1.4%)
Ischemic Stroke	4 (0.7%)
Hearing Loss	2 (0.4%)
Pulmonary Embolism	2 (0.4%)
Sepsis	2 (0.4%)
Others	4 (0.7%)
Severe Post-Radiotherapy Complications (*n* = 818)	No. (%)
Total	251 (30.7%)
Hypopituitarism	126 (15.4%)
Hearing Loss	58 (7.1%)
Radiation Necrosis	30 (3.7%)
Cranial Nerve Neuropathies	14 (1.7%)
Osteoradionecrosis	7 (0.9%)
ntracranial Hemorrhage	4 (0.5%)
Memory Loss	4 (0.5%)
Seizure	4 (0.5%)
Brainstem Gliomas	2 (0.2%)
Locoregional Brain Edema	2 (0.2%)
Otitis Media	2 (0.2%)
Convexity Meningioma	1 (0.1%)
Mucositis	1 (0.1%)
Supratentorial Glioblastoma	1 (0.1%)
Symptom Improvement (*n* = 316)	138 (46.7%)
Radiological Response (*n* = 247)	No. (%)
Reduced tumor volumes	67 (27.1%)
Stable tumor volumes	144 (58.3%)
ncreased tumor volumes	36 (14.6%)
Recurrence (*n* = 1307)	No. (%)
Local Recurrence	211 (16.1%)
Distant Metastases	7 (0.5%)
Treatment for Local Recurrence (*n* = 211)	No. (%)
Surgical resection alone	33 (15.6%)
Radiotherapy alone	29 (13.7%)
Surgical resection + Radiotherapy	17 (8.1%)
Stereotactic radiosurgery alone	11 (52%)
Proton therapy alone	4 (1.9%)
Surgical resection + Stereotactic radiosurgery	3 (1.4%)
Radiotherapy + Carbon therapy	1 (0.5%)
Radiotherapy + Stereotactic radiosurgery	1 (0.5%)
No treatment	25 (11.8%)
Not reported	87 (41.2%)
Outcome (months)	Median, range
Follow-up (*n* = 1307)	67, 0.1–376
Progression Free Survival (*n* = 159)	36, 0.1–300
5-year rate	84.3%
10-year rate	67.4%
Overall Survival (*n* = 233)	67, 0.1–376
5-year rate	94%
10-year rate	84%
Status (*n* = 1192)	No. (%)
Alive	1058 (88.8%)
Dead	134 (11.2%)

## Supplementary Materials

The following are available online at www.mdpi.com/2072-6694/13/23/5960/s1, Table S1: Overview of all included studies, Table S2: Risk of bias assessments for all included studies.
